# Draft Genome Sequence of *Streptococcus canis* Clinical Strain TA4, Harboring the M-Like Protein Gene and Isolated in Japan from a Patient with Bacteremia

**DOI:** 10.1128/genomeA.01469-17

**Published:** 2018-01-18

**Authors:** Haruno Yoshida, Yukie Katayama, Yasuto Fukushima, Daisuke Taniyama, Yoshiteru Murata, Tetsuya Mizutani, Takashi Takahashi

**Affiliations:** a Laboratory of Infectious Diseases, Kitasato Institute for Life Sciences & Graduate School of Infection Control Sciences, Kitasato University, Minato-ku, Tokyo, Japan; b Research and Education Center for Prevention of Global Infectious Diseases of Animals, Tokyo University of Agriculture and Technology, Fuchu, Tokyo, Japan; c Department of General Internal Medicine, Tokyo Saiseikai Central Hospital, Minato-ku, Tokyo, Japan

## Abstract

*Streptococcus canis* is an animal-origin β-hemolytic bacterium that can cause severe infections in animals and occasionally infects humans. Here, we report a draft genome sequence of an *S. canis* strain harboring the M-like protein gene. This strain was isolated from a patient with bacteremia (reported by Taniyama et al. [D. Taniyama, Y. Abe, T. Sakai, T. Kikuchi, and T. Takahashi, IDCases 7:48–52, 2017, https://doi.org/10.1016/j.idcr.2017.01.002]). The draft genome comprises 2,129,080 bp in 60 contigs.

## GENOME ANNOUNCEMENT

The emerging zoonotic pathogen *Streptococcus canis* was first reported in 1986 (1). This microorganism can sometimes cause self-limiting dermatitis and severe illnesses, such as arthritis, streptococcal toxic shock syndrome, necrotizing fasciitis, septicemia, and pneumonia, in companion and other animals with underlying diseases (2–4). *S. canis* can also infect humans who may or may not have been in close contact with animals and cause either local (ulcer infection) or systemic (septicemia or endocarditis) diseases (5–7). The *S. canis*-derived M-like protein (Scm), similar to the M protein in *Streptococcus pyogenes*, is an *S. canis* virulence factor capable of binding to plasminogen and immunoglobulin G, activating an antiphagocytic function in the hosts (8–10).

Eichhorn et al. (11) described a draft genome sequence of an Scm-positive *S. canis* strain (G361) isolated from a vaginal swab of a 40-year-old woman. We reported a patient in Japan who developed bacteremia following a bite by the pet dog and isolated a bacterial strain from the patient that contained an Scm gene (GenBank accession number LC228777), which was similar to those of two other *S. canis* strains, 321 324 A and 341 4291B (GenBank accession numbers KF662395 and KF662396, respectively) (12).

The *S. canis* strain TA4 (12), of human blood origin, was grown in Todd-Hewitt broth supplemented with yeast extract (THY broth) overnight. Genomic DNA was extracted using a DNeasy blood and tissue kit (Qiagen, Hilden, Germany) after pretreatment with lysozyme and proteinase K. A DNA sequencing library was prepared using a Nextera XT DNA sample prep kit (Illumina, Inc., San Diego, CA, USA) according to the manufacturer’s instructions. The library was indexed and sequenced using an Illumina MiSeq benchtop sequencer system with unrelated sequencing libraries.

Sequencing yielded 4,752,210 reads (810,126,104 bp), and the genome was assembled *de novo* using CLC Genomics Workbench (version 6.5.1). The assembled genome consisted of 60 contigs (GenBank accession numbers BEWZ01000001 to BEWZ01000060, ranging from 630 bp to 218,482 bp, respectively) with an average coverage of 372.1× and an *N*_50_ of 95,904 bp. The draft genome sequence was automatically annotated using the Microbial Genome Annotation Pipeline (http://www.migap.org) (13, 14). The total length of the TA4 genome was 2,129,080 bp (with a GC content of 39.7%), and it contained 2,040 coding sequences, 38 tRNAs, 3 rRNA loci, 1 prophage region, and 2 incomplete phage elements.

The mapping procedure in the comparative genome analysis revealed that 92.82% of the reads from the sequencing of TA4 were located on the complete genome sequence of *S. canis* strain FSL Z3-227 (GenBank accession number NZ_AIDX01000001), a milk-origin isolate from a cow with an intramammary infection (15). We attempted *de novo* assembly using the remaining 341,258 reads, and 47 contigs (GenBank accession numbers BEWZ01000061 to BEWZ01000107) were obtained. Many of the coding regions in these 47 contigs were found to encode phage-derived proteins that were highly homologous with phage and phage-associated proteins present in pathogenic streptococci (i.e., *S. pyogenes* serotypes M1, M2, M5, M6, M12, M28, and M53 and other streptococci). This finding suggests genetic transmission between the animal pathogen *S. canis* and the human pathogen *S. pyogenes*.

### Accession number(s).

The draft genome sequence of this strain has been registered in the DDBJ database under GenBank accession numbers BEWZ01000001 to BEWZ01000107.
